# Haematoma scavenging in intracerebral haemorrhage: from mechanisms to the clinic

**DOI:** 10.1111/jcmm.13441

**Published:** 2017-12-26

**Authors:** Gaiqing Wang, Li Wang, Xin‐gang Sun, Jiping Tang

**Affiliations:** ^1^ The second Hospital of Shanxi Medical University Tai Yuan China; ^2^ Department of Physiology Loma Linda University Loma Linda CA USA

**Keywords:** haematoma resolution, haematoma scavenge, scavenger receptors, intracerebral haemorrhage, neural recovery

## Abstract

The products of erythrocyte lyses, haemoglobin (Hb) and haem, are recognized as neurotoxins and the main contributors to delayed cerebral oedema and tissue damage after intracerebral haemorrhage (ICH). Finding a means to efficiently promote absorption of the haemolytic products (Hb and haem) around the bleeding area in the brain through stimulating the function of the body's own garbage cleaning system is a novel clinical challenge and critical for functional recovery after ICH. In this review, available information of the brain injury mechanisms underlying ICH and endogenous haematoma scavenging system is provided. Meanwhile, potential intervention strategies are discussed. Intracerebral blood itself has ‘toxic’ effects beyond its volume effect after ICH. Haptoglobin–Hb–CD163 as well as haemopexin–haem–LRP1 is believed to be the most important endogenous scavenging pathway which participates in blood components resolution following ICH. PPARγ–Nrf2 activates the aforementioned clearance pathway and then accelerates haematoma clearance. Meanwhile, the scavenger receptors as novel targets for therapeutic interventions to treat ICH are also highlighted.

**Table:** 

**• Introduction**
**• Current understanding of the mechanisms underlying ICH‐induced brain injury**
**• Phagocytosis in haematoma resolution**
**• Potential endogenous haematoma scavenger receptors after ICH**
**• CD36**
**• CD47**
**• SRA**
**• Hp‐Hb‐CD163**
**• Hx‐Heme‐LRP1**
**• The upstream regulatory mechanism and intervention strategy for scavenger receptors after ICH**
***‐*** **Nuclear factor erythroid 2‐related factor 2 (Nrf2)**
***‐*** **Peroxisome proliferator‐activated receptor‐γ (PPAR‐γ)**
**• Potential treatment options/strategies for haematoma resolution ** ***via*** **CD36**
***‐*** **PPAR‐γ agonists**
***‐*** **Nrf2 agonists**
**• The agonists for the other scavenger receptors**
***‐* CD163 agonists**
***‐* Haptoglobin(Hp) and haemopexin(Hx) agonists**
***‐* LRP1 agonists**
***‐* SRA agonists**
***‐* CD47 agonists**
***‐*** **Iron chelators**
***•*** **Conclusion**
***•*** **Acknowledgements**
***•*** **Conflict of interest**

## Introduction

Extravasated blood and subsequent intrahaematoma haemolytic products trigger a series of adverse events after intracerebral haemorrhage (ICH), leading to secondary brain injury, oedema and severe neurological deficits or death. Haematomas are the primary cause of neurological deficits associated with ICH. Although the haematoma in human's brain gradually resolves within months, full restoration of neurological function can be slow and often incomplete, leaving survivors with devastating neurological deficits. Unless haematoma is cleared, the reservoirs of blood continue to inflict injury to neurovascular structures and blunt the brain repair processes 1. However, only a few evidence‐based targeted treatments are used for ICH management, and interventions focus primarily on supportive care and comorbidity prevention. Effective haematoma clearance and/or facilitating haematoma absorption result in the removal of all the toxic components, which is a goal and a novel therapeutic strategy for ICH, as haematoma removal/resolution can relieve mechanical compression, limit inflammatory injury and promote the recovery of neuronal function 2, 3, 4.

Endogenous garbage cleaning system also known as scavenger receptors plays important roles in the regulation of haematoma resolution in ICH 5. This review seeks to understand how the endogenous garbage cleaning system or scavenger receptor system in the brain works together to remove blood from the brain and reduce brain damage, then find a medical measure to speed up this process.

## Current understanding of the mechanisms underlying ICH‐induced brain injury

**Figure 1:**
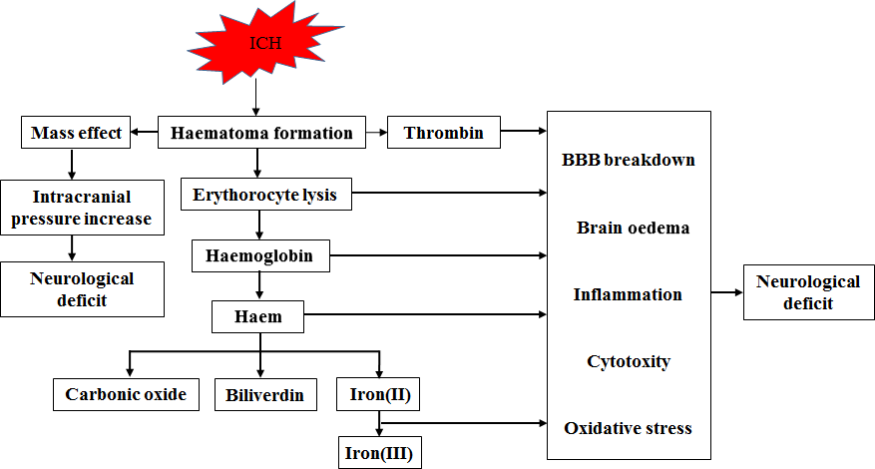
The major factors contributing to brain injury after ICH (including mass effect, thrombin and blood components).

**Figure 2:**
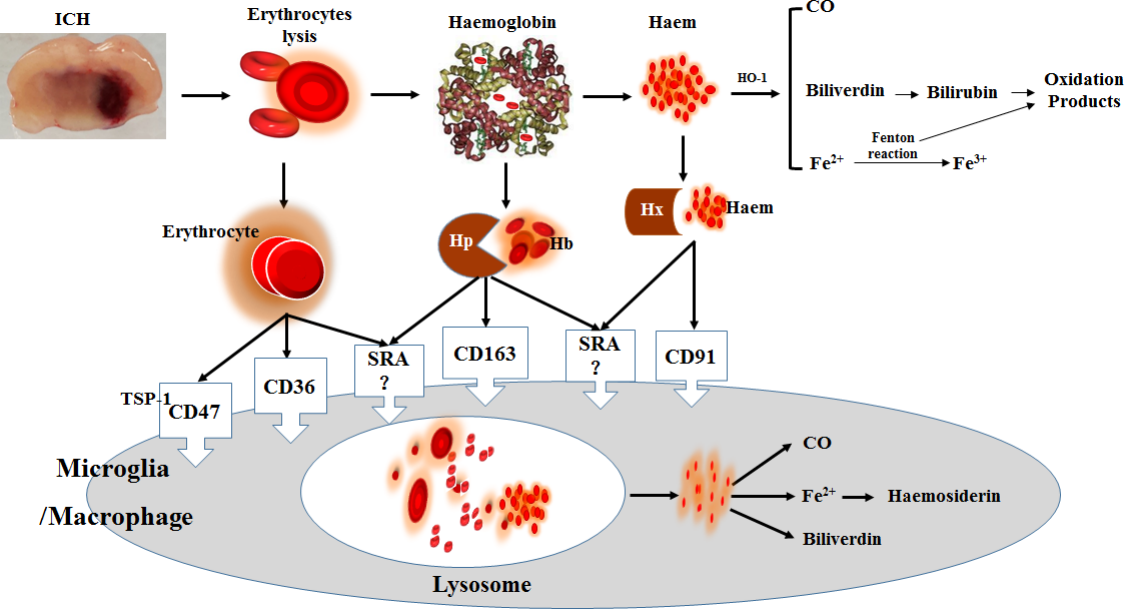
The potential endogenous scavenger receptors (such as CD36, CD47, SRA, Hp–Hb–CD163 and Hx–haem–CD91) following ICH.

Brain injury due to ICH initially occurs within the first few hours as a result of mass effect due to haematoma formation. But many patients continue to deteriorate clinically despite no signs of rehaemorrhage or haematoma expansion. This continued insult after primary haemorrhage is believed to be mediated by direct toxicity and inflammatory responses induced by the components and metabolic products of late‐stage haematomas and aggravates neurological deficits 6. In other words, intracerebral blood itself has ‘toxic’ effects beyond its mass effect 7. Oxidative stress caused by components of the lysed erythrocytes contributes to the brain injury after ICH 8. Hb, haem and iron released after red blood cell lysis aggravate ICH‐induced severe brain oedema and direct neuronal damage(Fig. 1). To offset this process, phagocytic cells, including the brain's microglia and haematogenous macrophages, phagocytose and then remove extravasated erythrocytes before lysis and subsequent toxicity occurs 3. So the better understanding of phagocytosis through corresponding scavenger receptors is beneficial to explore removal of blood from the ICH‐affected brain, thus limiting/preventing haemolysis from occurring 5. The potential endogenous scavenger receptors following ICH were illustrated in Figure 2.

## Phagocytosis in haematoma resolution

Microglia/macrophages (MMΦ) represent the primary phagocytic system and the first line of defence against brain injuries that mediates the clean‐up of haematoma. Thus, the efficacy of phagocytic function by microglia/MMΦ is an essential step in limiting ICH‐mediated damage 1. The resident microglia and peripheral macrophages are rapidly mobilized to the lesions and initiate the release of mediators and recruitment of other immune cells 9. Microglia is a key factor to remove the haematoma and clear debris, but it is a source of ongoing inflammation. Activated microglia/MMΦ may play a potentially detrimental neurotoxic role by eliciting the expression of pro‐inflammatory and initiating neuroprotective properties. Although overactivation of microglia/MMΦ amplifies inflammatory neuronal damage, anti‐inflammatory agents have failed to show clinical benefits in many stroke trials so far. The undesired effects may result from a broad suppression of microglia/ MMΦ which deprive the normal defensive functions of brain. It is worth noting that acute inflammation serves many protective functions, whereas chronic inflammation is more likely to exacerbate injury. So experimental stroke therapies should be shifted from blanketed microglia/ MMΦ suppression towards a more nuanced adjustment of the balance between protective and toxic microglia/MMΦ phenotypes 10. Phagocyte activation inevitably release pro‐inflammatory mediators and free radicals during haematoma resolution which is toxic to neighbouring cells, leading to secondary brain injury, but promotion of phagocytosis in a timely and efficient manner which may limit the toxic effects of persistent blood products on surrounding tissue and this manner may be important for recovery after ICH 2. The process of the haematoma resolution probably be related to the concomitant acute brain swelling. Recent studies found that enhancing microglia/MMΦ‐mediated phagocytosis speeds up haematoma clearance and then improves functional outcome after ICH 1. Scavenger receptors, expressed on microglia/astrocytes or endothelial cells, play important roles in the regulation of phagocytosis in microglia/MMΦ.

## Potential endogenous haematoma scavenger receptors after ICH

As shown in Figure 2, potential endogenous scavenger receptors, as a major subset of innate pattern recognition receptors, are mainly functioned in endocytosis and exogenous invaders 11. They play a crucial role in maintenance of cerebral homeostasis and phagocytic regulation. This section summarizes the newly recognized functions of scavenger receptors in haematoma clearance following ICH.

## CD36

CD36 is a well‐recognized integral microglia/macrophage cell membrane protein and a type II scavenger receptor which is expressed on the surface of macrophages and monocytes and plays an important role in mediating the recognition and phagocytosis. Cells lacking phagocytic abilities acquire phagocytic functions following transfection with CD36 12. The low levels of CD36 present may control adhesion of erythrocytes and may have a signal transduction role in platelets and monocytes. CD36 and thrombospondin (TSP) appear to be involved in several cell adhesion processes, including thrombin‐induced platelet aggregation, adhesion of platelets to monocytes and so on 13.

## CD47

CD47 is an integrin‐associated transmembrane protein expressed in a variety of cells types including microglia/ MMΦ, oligodendrocytes and erythrocytes. CD47, a well‐known ‘don’t eat me' signal, controls erythrocyte lifespan positively through inhibition of phagocytosis *via* signal regulatory protein (SIRPα) on normal/healthy erythrocytes, and it revealed an important role in the clearance of aged erythrocytes 14. CD47 on erythrocytes and other cells can function as a regulator of target cell phagocytosis 15. As a switch for erythrophagocytosis, CD47 undergoes a conformational change during ageing, which causes thrombospondin (TSP‐1) binding and recognition of CD47 as an ‘eat me’ signal by SIRPα. The conformational status of CD47 can be changed through oxidative stress, and binding of TSP‐1 to apoptotic cells enhanced phagocytosis without inducing the secretion of pro‐inflammatory cytokines 14. CD47 expression was increased in the perihaematomal white and grey matter after ICH, and deferoxamine treatment attenuated brain CD47 expression after ICH 16. Higher microglial activation at day 3 after experimental ICH was found after CD47 knockout blood injection, and CD47 has a key role in haematoma clearance after ICH 17. The present results of CD47 in ICH are confusing and are contradictory to its phagocytic property in pathological conditions, and the exact role of CD47 in erythrocyte clearance after ICH still needs to be further study.

## SRA

Scavenger receptor A (SRA), also known as the macrophage scavenger receptor and cluster of differentiation 204 (CD204), plays roles in lipid metabolism, atherogenesis and a number of metabolic processes. SRA is reported to be host protective in some disease states, but there is also compelling evidence that SRA plays a role in the pathophysiology of other diseases 18, 19. On the one hand, SRA is clearly beneficial and host protective in some models of disease. SRA on microglial cells mediates the binding of β amyloid fibrils and is responsible for preventing the accumulation of amyloid in the brain. The decrease in SRA activity could contribute to the progression of neurodegeneration 20. SRA was highly expressed in erythrocyte lysate‐treated microglia. Genetic SRA ablation increased microglia activation and cytokine production, and sensitized mice to ICH‐induced neuron injury 21. SRA down‐regulated inflammatory response expression in microglia by suppressing TLR4‐induced activation 22. SRA mediates activation of inflammatory signalling and apoptosis in ischaemic stroke, both of which contribute to cerebral injury. The published data have given rise to an intriguing dilemma. As well as the other markers of microglia/MMΦ activation, SRA is a two‐edged sword in health and disease 18, 19. Oxidized erythrocytes were internalized *via* SRA on macrophages and then sent to lysosomes for scavenging. The exact role of SRA in ICH is dramatic and unclear, as a scavenger receptor, and it should participate in haematoma resolution through microglia activation and maybe produce undesirable inflammatory response, but the results of SRA in ICH are confusing 21, 22.

## Hp–Hb–CD163

CD163 is a phagocytic marker and a haemoglobin scavenger receptor, of which expression is thought to be exclusive to perivascular (PVM) and monocyte—macrophage system. It is a glycoprotein belonging to class B of the scavenger receptor cysteine‐rich superfamily. It functions as a membrane‐bound scavenger receptor for clearing extracellular haptoglobin–haemoglobin (Hp‐Hb) complexes 23. Both *in vitro* and *in vivo* investigations have shown that ROS is highly produced after exposing Hb to cell culture or injecting Hb into mouse striatum 24, 25. Haptoglobin(Hp) is the primary Hb‐binding protein in human plasma, which attenuates the adverse biochemical and physiological effects of extracellular Hb. The cellular receptor target of Hp is the monocyte/MMΦ scavenger receptor, CD163. Excessive Hb up‐regulated expression of Hp and the Hb–Hp receptor CD163 in neurons *in vivo* and *in vitro* 26. Free Hb binds to Hp and once Hp‐Hb complex is endocytosed by CD163, which mediated delivery of Hb to the macrophage may fuel an anti‐inflammatory response because haem metabolites have potent anti‐inflammatory effects 27.

## Hx–Haem–LRP1

With chronic haemolysis following ICH, Hp is depleted and Hb readily distributes to tissues where it might be exposed to oxidative conditions. In such conditions, haem can be released from ferric Hb. The free haem is highly toxic which can accelerate tissue damage by promoting peroxidative reactions and activation of inflammatory cascades 28. The haem scavenger protein–haemopexin (Hx) contributes to haematoma removal as well as Hp‐Hb after ICH 29, and Hx is another plasma glycoprotein able to bind haem with high affinity. Hx sequesters haem in an inert, non‐toxic form and transports it to the liver for catabolism and excretion 30. Hp and Hx have been characterized as a sequential defence system with Hp as the primary protector and Hx as a backup when Hp has been depleted during severe ICH. The linear relationship between Hx concentration and protection defined a highly efficient backup scavenger system during conditions of large excess of free Hb 31. The haem–Hx complex is endocytosed by cells expressing the low‐density lipoprotein receptor‐related protein‐1 (LRP1)/CD91 receptor 32, 33. LRP1 is a transmembrane receptor expressed on several cell types including macrophages, hepatocytes, neurons, vascular endothelial cells, pericytes, smooth muscle cells and astrocytes. Half of the BBB clearance is mediated by brain endothelial LRP1 in various model systems 34. As the only known endocytic receptor for Hx–haem complexes, LRP1 combined function of Hx may mediate localized haem clearance in the brain during cerebral haemorrhage. Upon binding of haem–Hx to LRP1, the complex becomes internalized *via* endocytosis into cells, and inside the cell, the haem–Hx complex is dissociated by lysosomal activity. Haem is catabolized by haem oxygenases into biliverdin, carbon monoxide and iron. Activation of LRP1 scavenging system in humans has favourable effects after subarachnoid haemorrhage (SAH) 35. Recently, it is confirmed that the activation of the LRP1 system is beneficial in experimental ICH 33. It should be proved in clinic through above‐mentioned findings.

## The upstream regulatory mechanism and intervention strategy for scavenger receptors after ICH

### Nuclear factor erythroid 2‐related factor 2 (Nrf2)

**Figure 3:**
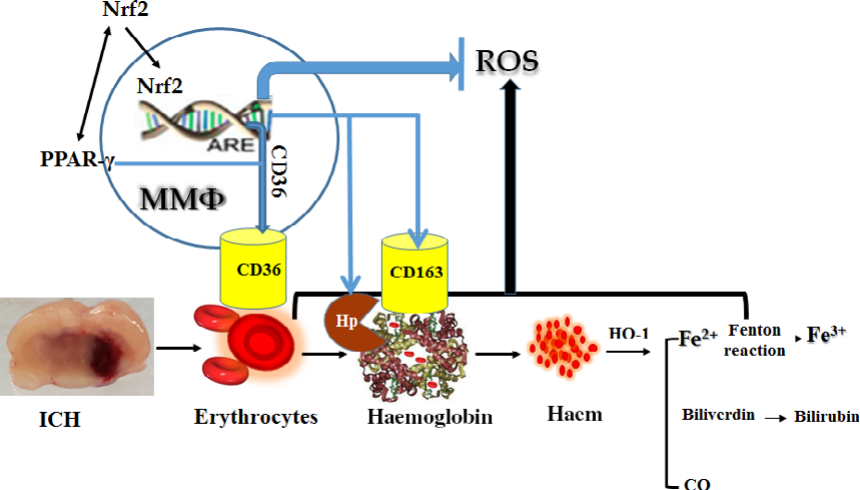
The potential role of Nrf2 and the interaction of PPAR‐γ with Nrf2 following ICH.

Nrf2 itself is a ubiquitous pleiotropic transcription factor and a pivotal mediator in redox homeostasis and inflammatory disorders, within the regulated region of many cytoprotective and antioxidant target genes which encode for critical mediators of cellular defence functions 36. In unstressed conditions, Nrf2 is retained in the cytoplasm by its inhibitor kelch‐like ECH‐associated protein 1 (Keap1), upon activation by oxidative and electrophilic stress, and Nrf2 disassociates from Keap1, transactivates the antioxidant response element (ARE) and then promotes the related cytoprotective pathways. So Nrf2 is a key regulator of cellular resistance against oxidants 37. In addition, the transcriptional activity of Nrf2 is essential for the clearance of phosphorylated tau *via* the selective autophagy 38. Also, knockout of Nrf2 reduced the efficiency of macrophage accumulation and impaired clearance of myelin debris and phosphorylated tau 39. Nrf2 controls the expression of CD36, which may represent a key component in attaining brain clean‐up after stroke or ICH. Removal of RBC by microglia and/or MMΦ may have a multiple indirect effect on oxidative stress, as it could reduce the accumulation of haemoglobin–haem–free iron and consequently the formation of free radicals. Furthermore, activated Nrf2 up‐regulated the levels of Hp in blood plasma and in ICH‐affected brain in animals as well as the expression of CD163 40. In conclusion, Nrf2 in microglia/MMΦ plays a pivotal role in regulating the phagocytic functions of these cells, and that in an experimental model of ICH, Nrf2 appears to be essential to haematoma clearance 1. The potential role of Nrf2 following ICH is illustrated in Figure 3.

### Peroxisome proliferator‐activated receptor‐γ (PPAR‐γ)

The peroxisome proliferator‐activated receptor‐γ (PPAR‐γ) is a ligand‐activated transcription factor belonging to the nuclear hormone receptor superfamily and controlling reproduction, metabolism, development and immune response. PPAR‐γ expressed not only in adipocytes, but also in vascular tissues, such as vascular smooth muscle cells (VSMCs) and endothelial cells, and in macrophages 41. PPAR‐γ and its agonists have a protective role in several neurological diseases *via* reducing inflammation, decreasing oxidative damage and attenuating cell death 42. PPAR‐γ is protective not only to neurons, astrocytes, oligodendrocytes, endothelia, but also to microglia/MMΦ both *in vitro* and *in vivo*. PPAR‐γ agonists reduce the ability of inflammatory stimuli to activate the alveolar macrophage while simultaneously stimulating phagocytosis of both opsonized and unopsonized particles, *via* the Fc‐γ and CD36 receptors, respectively 43. PPAR‐γ ligands have also been shown to up‐regulate the expression of CD36 and then promote microglia/MMΦ‐mediated clearance of toxic cellular debris 44. PPAR‐γ agonists not only increased microglia‐mediated phagocytosis of RBC, but also reduced the production of H_2_O_2_ during the process of engulfment 2, 44. So enhancement of phagocytosis by PPAR‐γ agonists inevitably results in the inflammatory response and the dose‐dependent neurotoxicity.

**Figure 4:**
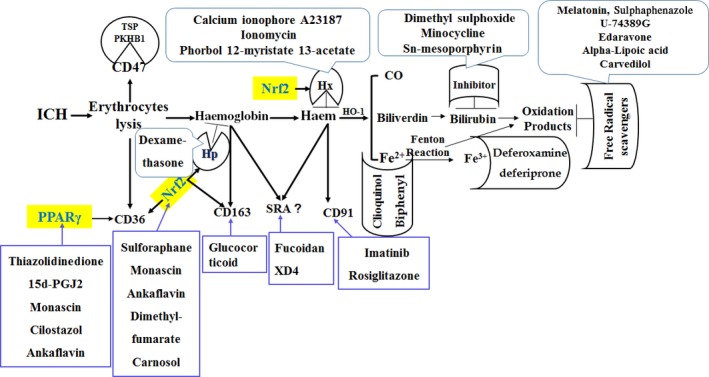
Summary of current potential exogenous pharmacological/molecular manipulations direct at haematoma resolution after ICH.

Some studies showed PPAR‐γ had an interaction with Nrf2. Endogenous PPAR‐γ ligands activate Nrf2 expression, meanwhile Nrf2 regulated PPAR‐γ expression 45. Nrf2 controls CD36 expression independently of PPAR‐γ. Expression of Nrf2 was reduced by knockdown of PPAR‐γ, whereas PPAR‐γ was reduced by knockdown of Nrf2, thereby demonstrating two‐way positive interactions. PPAR‐γ agonists up‐regulate Nrf2, and knockdown of PPAR‐γ reduced the mRNA expression for Nrf2 46. This indicates a tight, positive, two‐way reinforcing transcriptional interaction between PPAR‐γ and Nrf2 that may improve endothelial function 46. The interaction of PPAR‐γ with Nrf2 following ICH was illustrated in Figure 3. Potential exogenous pharmacological/molecular manipulations direct at haematoma resolution after ICH (as illustrated in Fig. 4).

## Potential treatment options/strategies for haematoma resolution *via* CD36

### PPAR‐γ agonists

PPAR‐γ agonist‐induced up‐regulation of CD36 in macrophages enhances the ability of microglia to phagocytose red blood cells (*in vitro* assay), helps to improve haematoma resolution and reduces a mouse ICH‐induced deficit. In rat primary microglia in culture, PPAR‐γ agonists not only increased microglia‐mediated phagocytosis of RBC, but also reduced the production of H2O2 during the process of engulfment 2. PPAR‐γ agonists could represent a potential treatment strategy for ICH 3.

Thiazolidinediones are potent and selective activators of PPAR‐γ 47. The derivatives of the parent compound thiazolidinedione such as rosiglitazone and pioglitazone are generally well tolerated in AD and aMCI patients 48. The Safety of Pioglitazone for Hematoma Resolution In ICH (SHRINC) Study 49, a prospective, randomized, blinded, placebo‐controlled, dose‐escalation safety trial, was recently completed, and its results showed that pioglitazone should be a potential therapy for ICH in future. The cyclopentanone prostaglandins (*e.g*. 15d–PGJ2) and monascin as well as thiazolidinediones are PPAR‐γ agonists, which have been proven to act as potent and safe pro‐survival factors for primary neurons subjected to either excitotoxic insult, oxygen–glucose deprivation (OGD) or H2O2‐induced oxidative stress 44, 47, 50 and then attenuate ROS generation. These PPAR‐γ agonists are proposed to act as endogenous PPAR‐γ ligands demonstrate rather limited selectivity towards PPAR‐γ with some of its biological activation of Nrf2. Monascin regulated PPAR‐γ and Nrf‐2 to improve lung oxidative inflammation 51. Cilostazol, a potent phosphodiesterase type III inhibitor, has been used as a vasodilating antiplatelet drug for the treatment of ischaemic symptoms in chronic peripheral arterial obstruction for preventing recurrence of cerebral infarction 52. Cilostazol stimulates PPAR‐γ transcriptional activity in human endothelial cells and may offer an effective therapeutic window along with complementary effects for individuals at high risk of type 2 diabetes by improving insulin sensitivity with anti‐inflammatory effects 53. Ankaflavin exerted PPAR‐γ agonist activity by the up‐regulation of the signalling pathway of Nrf2 54.

The existing evidence showed that dose‐dependent neurotoxicity of the 15d–PGJ2 in cerebellar granule cells, primary cortical neurons and spinal cord motor neurons which were believed to be associated with induction of apoptosis and not likely associated with the activation of PPAR‐γ 55. On the other hand, the clinically relevant, more selective PPAR‐γ agonist, such as rosiglitazone, was linked to peripheral oedema, increase in body weight, and cardiomyopathies and heart failure 56. It is likely that PPAR‐γ agonist treatment for ICH will be short term, potentially avoiding these side‐effects, although this needs further testing.

### Nrf2 agonists

Nrf2 as a second important transcription factor involved in the induction of the scavenger receptor CD36 and antioxidant stress genes in atherosclerosis 57. Nrf2 transcription factor could be an alternative target to PPAR‐γ in the control of severe malaria through parasite clearance 58. Nrf2 plays an essential role in the effective clean‐up process after ICH, perhaps *via* co‐ordinated efforts to enhance phagocytosis while concomitantly limiting oxidative stress 1. Sulforaphane was capable of enhancing RBC phagocytosis and improving haematoma resolution *via* activating Nrf2 and inducing CD36 expression in microglias 1. Monascin acts as a novel natural Nrf2 activator with PPAR‐γ agonist activity was confirmed by Nrf2 and PPAR‐γ reporter assays 59. Protective effects of ankaflavin against diabetes are mediated by the up‐regulation of the signalling pathway of Nrf2, which enhances antioxidant activity and serves as a PPAR‐γ agonist to enhance insulin sensitivity 54. Monascin and ankaflavin, the yellow pigments produced by Monascus species, have been proven to possess hypolipidaemic functions and less side‐effects 60. Dimethyl fumarate is an orally administered fumarate ester recently FDA approved for first‐line monotherapy of multiple sclerosis, which stimulates Nrf2 activity to attenuate hyperphosphataemia *in vitro* or vitamin D3‐induced *in vivo* vascular calcification 61. Carnosol increased the nuclear levels of Nrf2 and involved in the cytoprotective effects 62.

## The agonists for the other scavenger receptors

### CD163 agonists

To date, the Hb–haptoglobin (Hp) complex is the only known ligand of CD163, and neither Hp alone nor free Hb has been found to display high‐affinity binding to the receptor. Because the Hb–Hp complex binds to CD163 with high affinity and the receptor system has a high endocytotic capacity, CD163 is thought to mediate the clearance of Hb–Hp complexes from the blood 27. Glucocorticoid can induce CD163 expression in MMΦ which enhances their capacity to bind and internalize Hb–Hp complexes 63.

### Haptoglobin(Hp) and haemopexin(Hx) agonists

Hp expression is induced by inflammatory cytokines, dexamethasone and adrenoceptor agonists. In contrast, Hp was inhibited by nicotinic acid and the PPARγ agonist, rosiglitazone 64. The transcription rate of Hx increased by the calcium ionophore A23187, ionomycin and phorbol 12‐myristate 13‐acetate (PMA) in serum‐starved H4IIE rat hepatoma cells 65. Activated Nrf2 binds to antioxidant response elements (ARE), which promotes the transcription of haptoglobin and haemopexin 66.

### LRP1 agonists

LRP1/CD91 contributed to haem clearance and blood–brain barrier protection after ICH in mice. Our research showed that recombinant LRP1 as supplement provides a novel approach to ameliorate intracerebral haemorrhage brain injury *via* its pleiotropic neuroprotective effects 33. Intrathecal infusions of LRP1 agonists—RBD (the Receptor Binding Domain of alpha‐2‐macroglobulin) or PEX (the haemopexin domain of MMP‐9) result in axonal sprouting and regeneration after spinal cord injury *via* activating ERK and Akt pathways 67. Imatinib promotes LRP1‐dependent ERK activation and helps to the pro‐survival effects on β‐cells 68.

### SRA agonists

Fucoidan, a SRA agonist, could promote macrophage apoptosis by repressing ER stressor triggered autophagy 69. Heptapeptide XD4 activates SRA on the glia by increasing the binding of Aβ to SRA, thereby promoting glial phagocytosis of Aβ oligomer in microglia and astrocytes 70.

### CD47 agonists

CD47 stimulated by its ligands, thrombospondins (TSPs), the agonist sequences occur in all five isoforms of TSP, it is possible that any TSP isoform could be pro‐apoptotic 71. PKHB1, the serum‐stable CD47 agonist peptide, might overcome drug refractoriness of chronic lymphocytic leukaemia by the pro‐apoptotic potential of targeting cell 72.

### Iron chelators

Our study showed that ferric iron chelators such as deferoxamine and deferiprone lowered iron deposition in brain following ICH 73. However, ferric iron chelation does not improve the outcome after ICH 33, 74. Clioquinol, a ferrous iron chelator, improved the neurological outcome and attenuated brain oedema and ROS production besides reducing iron levels 73. Another ferrous chelator, 2,2′‐bipyridine, is a potential means of ameliorating iron‐induced injury after ICH 75; unfortunately, another results failed to support the use of bipyridine against ICH 76, and the function of bipyridine on ICH is uncertain so far.

## Conclusion

Hp–Hb–CD163 as well as Hx–haem–LRP1 is believed to be the most important endogenous garbage scavenging pathway which participates in haematoma/ blood components resolution following ICH. PPARγ–Nrf2 activates the aforementioned clearance pathway and then accelerates haematoma removal. So the above‐mentioned haematoma scavenger pathway as a novel targets for therapeutic interventions to treat ICH is prospective and valuable.

## Conflict of interest

The authors confirm that there is no conflict of interests.
